# Reducing the Burden of Anemia and Neural Tube Defects in Low- and Middle-Income Countries: An Analysis to Identify Countries with an Immediate Potential to Benefit from Large-Scale Mandatory Fortification of Wheat Flour and Rice

**DOI:** 10.3390/nu13010244

**Published:** 2021-01-16

**Authors:** Vijaya Kancherla, Manpreet Chadha, Laura Rowe, Andrew Thompson, Sakshi Jain, Dylan Walters, Homero Martinez

**Affiliations:** 1 Center for Spina Bifida Prevention, Department of Epidemiology, Emory University Rollins School of Public Health, Atlanta, GA 30322, USA; vijaya.kancherla@emory.edu; 2 Nutrition International, Ottawa, ON K2P 2K3, Canada; athompson@nutritionintl.org (A.T.); sjain@nutritionintl.org (S.J.); dwalters@nutritionintl.org (D.W.); hmartinez@nutritionintl.org (H.M.); 3 Food Fortification Initiative, Atlanta, GA 30322, USA; laura.rowe@ffinetwork.org

**Keywords:** DALYs, fortified foods, developing countries, neural tube defects, anemia, child mortality, infant mortality, women’s health, Sustainable Development Goals, health policy

## Abstract

Using a predetermined set of criteria, including burden of anemia and neural tube defects (NTDs) and an enabling environment for large-scale fortification, this paper identifies 18 low- and middle-income countries with the highest and most immediate potential for large-scale wheat flour and/or rice fortification in terms of health impact and economic benefit. Adequately fortified staples, delivered at estimated coverage rates in these countries, have the potential to avert 72.1 million cases of anemia among non-pregnant women of reproductive age; 51,636 live births associated with folic acid-preventable NTDs (i.e., spina bifida, anencephaly); and 46,378 child deaths associated with NTDs annually. This equates to a 34% reduction in the number of cases of anemia and 38% reduction in the number of NTDs in the 18 countries identified. An estimated 5.4 million disability-adjusted life years (DALYs) could be averted annually, and an economic value of 31.8 billion United States dollars (USD) generated from 1 year of fortification at scale in women and children beneficiaries. This paper presents a missed opportunity and warrants an urgent call to action for the countries identified to potentially avert a significant number of preventable birth defects, anemia, and under-five child mortality and move closer to achieving health equity by 2030 for the Sustainable Development Goals.

## 1. Introduction

Globally, more than two billion people are not getting the micronutrients (vitamins and minerals) they need to survive and thrive. This includes two of the most critical micronutrients for human development—iron and folate (vitamin B9). Poor diet and limited access to nutrient-rich foods are common reasons for a person’s inadequate intake of micronutrients. Insufficient dietary intake of iron leads to iron-deficiency anemia (IDA)—the most widespread micronutrient disorder globally affecting nearly 1.6 billion people each year [1,2]. IDA impairs cognitive and motor development, causes fatigue, lowers productivity, and contributes to increased risk of death for a mother and her baby during pregnancy. However, due to limited data at the country level on IDA, anemia was used to estimate potential impact of iron fortification under the assumption that, globally, 50% of anemia may be caused by IDA, although evidence now suggests that this global figure should be context-specific [1].

Insufficient folate status in the periconceptional period (1–3 months prior to pregnancy and up to the first 28 days after conception) can result in severe and potentially fatal birth defects involving the closure of the neural tube, called neural tube defects (NTDs). In 2015, an estimated 260,100 (95% uncertainty interval: 213,800–322,000) pregnancies were affected by NTDs globally (i.e., approximately 20 cases per 10,000 births), and they largely comprise of spina bifida (~50%) and anencephaly (~38%), along with encephalocele (~13%) to a lesser extent. Of these affected pregnancies, about one in four resulted in elective termination of pregnancy and another one in four in stillbirths; 80% of the 143,200 live-born infants died before age five [3]. Children surviving with spina bifida face lifelong disabilities and require long-term care [4]. The prevalence of folic acid-preventable NTD cases can be reduced to a lowest achievable level of six cases per 10,000 births by ensuring adequate intake of foods enriched with folic acid (the synthetic form of naturally occurring folate) by women of reproductive age (WRA) during the critical periconceptional window [5].

Staple food enrichment or fortification is a low-cost, high-impact intervention [6] often targeted to medium- and large-scale milling practices and helps address a portion of a population’s dietary nutrient gaps and improve health status through the addition of context-specific micronutrient quantities to food staples. Food fortification is one complementary approach to addressing inadequate dietary intake of nutrients such as iron and folate. By adding essential micronutrients to staple foods regularly consumed by populations at large, a significant proportion of adverse health outcomes associated with micronutrient deficiency can be averted [7]. Importantly, this intervention does not require modification to a population’s eating habits, nor does it alter the taste, texture, or smell of staple foods; it is generally well accepted by consuming populations [8]. Food fortification is also highly cost-effective as the incremental cost of fortification for consumers is typically within the range of regular market-price fluctuations of up to 2% [8]. 

Wheat flour and rice have proven to be ideal vehicles for fortification in many countries due to their widespread coverage and high consumption throughout the year. These staples are also produced in large-scale mills, making them two of the most effective vehicles to deliver nutrients to large segments of the population. Large-scale fortification refers to fortification that happens at industrial, large-scale mills that produce on average ≥20 metric tons of wheat flour or rice per day. In mills of this size, fortification is considered practical and feasible [9]. Mandatory fortification of staple foods, as opposed to voluntary fortification, promotes compliance for domestic production in addition to imported goods, limits the amount of consumer behavior change required, ensures added nutrients can more effectively be measured and monitored, and promotes desirable health impacts due to increased population coverage compared to voluntary policy environments [10]. Mandatory programs have proven to be the most effective means of implementing large-scale fortification programs, as illustrated by a systematic review and meta-analysis of 179 studies, which showed that the total prevalence of spina bifida was 1.5 times lower in countries that had mandatory fortification programs in comparison to that found in countries where fortification was voluntary or had no fortification [11]. Currently, 85 countries around the world mandate the fortification of wheat flour and seven countries mandate the fortification of rice; additionally, 14 countries voluntarily fortify wheat flour and seven countries voluntarily fortify rice [12].

Ending micronutrient malnutrition, thereby addressing the nutritional needs of children, adolescent girls, and women, is an essential component of efforts to ensure countries have equal opportunities for increased productivity, reduced healthcare costs, and life-cycle improvements in health and wellbeing, as well as to achieve a number of the Sustainable Development Goals (SDG), including SDGs 1, 2, 3, 4, 5, 8, 10, 11, and 17 [13]. 

The objective of this paper is to identify low- and middle-income countries (LMICs) with a high potential for health and human capital impact through implementing large-scale fortification of wheat flour and/or rice with iron and/or folic acid on the basis of best available data as of December 2019. Among identified LMICs, we estimated: (1) country-specific and total prevalence of anemia averted in WRA if countries adopted large-scale fortification of wheat flour and/or rice with recommended levels and forms of iron, (2) country-specific and total number of pregnancies affected with folic acid-preventable NTDs averted if eligible countries adopted large-scale fortification of wheat flour and/or rice with recommended levels of folic acid, and (3) the potential economic value of the health benefits of fortification of wheat flour and/or rice with iron and/or folic acid at scale within the eligible LMICs.

## 2. Materials and Methods 

Our analysis was initially informed by global and country-specific leaders and partners currently engaged in tracking and promoting large-scale food fortification, with intimate knowledge of national wheat flour and rice fortification efforts and industrial milling opportunities. Country-specific data and information on fortification program status were compiled, vetted, and analyzed by authors collaborating from Nutritional International (NI), the Food Fortification Initiative (FFI), Emory University’s Center for Spina Bifida Prevention (CSBP), ReachAnother Foundation, and International Federation for Spina Bifida and Hydrocephalus (IF).

Country selection began with 76 countries suggested by the above collaborating partners on the basis of their knowledge of current fortification efforts in the countries, including appropriate vehicles and current milling infrastructure, political factors, and the presence of an enabling environment from both a public- and a private-sector perspective. Careful consideration was given to these suggestions due to the depth of experience these partners have across programs. Consideration was then given to the prevalence of country-specific anemia and NTDs and to the degree to which fortification would be a suitable means of addressing the burden in each country. The following criteria were then used to guide the prioritization of countries with the highest impact potential for improving the population’s health through mandatory fortification of wheat flour and/or rice with iron and/or folic acid: 

(1) *Prevalence of anemia among women 15–49 years is >30%.* As per the World Health Organization (WHO) classification [2], a prevalence of anemia over 30% among WRA indicates a moderate public health problem and a prevalence equal to or over 40% indicates a severe public health problem [14]. It is worth highlighting that only a few countries have data to differentiate between anemia and IDA. Due to this limitation, we relied on information about anemia prevalence, under the assumption that, globally, 50% of anemia may be due to IDA [2]. A recent systematic review suggests that this figure may be an overestimate and that actual prevalence of IDA should be assessed in a context-specific manner [1]; and/or.

*Prevalence of NTDs is >6 per 10,000 births.* A recent analysis using blood folate concentrations established that the minimum prevalence of NTDs at optimal blood folate concentrations is six per 10,000 births [5]. 

(2) *Per capita consumption of wheat flour/rice is ≥75 g/capita/day.* According to the WHO, estimated average per capita consumption levels <75 g per day do not allow for added micronutrients obtained through a fortification program to meet the nutritional needs of WRA [15]. Country-specific estimates of grain available per capita for consumption were sourced from the Global Fortification Data Exchange (GFDx) website [12]. The data for wheat flour availability includes wheat flour and a variety of wheat-based food products such as pasta, bread, biscuits, and breakfast cereals.

(3) *≥75% of the country’s wheat flour/rice is industrially milled.* We used data from FFI on the percentage of wheat flour/rice produced in each country that is industrially milled or that is imported from other countries according to responses to an FFI annual survey given to countries [16]. Industrially milled flour that is produced domestically or imported is considered to have the potential for immediate fortification in our analysis.

(4) *Optimal fortification design includes status of mandatory fortification or use of appropriate iron and/or folic acid levels and/or compounds*. This included countries that were not implementing a mandatory wheat flour/rice fortification program optimally or at all, countries that were implementing a program without iron or folic acid as a fortificant and where the burden of disease indicated a need for the nutrient, or countries implementing programs without iron or folic acid as a fortificant at the recommended level or in the recommended form per WHO guidelines [12]. For example, the Philippines has mandatory fortification of both wheat flour and rice but does not include folic acid in the standard. India is another such example where the policy guidance for fortification includes folic acid but at such a low level that it will not make an impact on reducing the prevalence of folic acid-preventable NTDs.

(5) <*50% of the industrially milled wheat flour/rice is fortified with iron and/or folic acid.* Country-specific coverage data for fortification of industrially milled wheat flour/rice was used [12]. Where these data could not be obtained, they were estimated on the basis of partner organization knowledge of the country context. The estimates ensure greater sensitivity in the identification of most countries with immediate fortification potential. 

Following the selection of countries based on criteria stated above, and according to the assessed potential of wheat flour/rice fortification at scale in each, we then estimated avertable cases of anemia, folic acid-preventable NTDs, under-five child deaths, disability-adjusted life years (DALYs), and the economic value of these avertable adverse health outcomes in each country. 

### 2.1. Estimation Method for Number of Anemia Cases Prevented in Eligible Countries

*Step 1: Quantifying proportion of wheat flour/rice that is industrially milled.* The proportion of wheat flour/rice that is industrially milled in a country was estimated by adding the domestic production of industrially milled wheat flour/rice with quantities of grains imported (assumes all imported wheat flour/rice is likely to be industrially milled) and subtracting exported grain, as well as post-harvest and post-processing losses.

*Step 2: Quantifying proportion of wheat flour/rice that is industrially milled but not currently fortified.* The current percentage of industrially milled wheat flour/rice that is fortified was compared that to the overall proportion of industrially milled wheat flour/rice in the country [12]; the difference represents the amount of wheat flour/rice that is industrially milled with the potential to be fortified.

*Step 3: Estimating the total population and total WRA that could be reached by fortified wheat flour/rice (percentage of population consuming the food vehicle)*. According to readily available per capita consumption data from United Nation’s Food and Agricultural Organization (FAO), total production potential estimated in the previous step was divided by country-specific consumption to quantify the total number of people that could be reached by large-scale fortification; this was stratified for specific demographics including WRA. This information was obtained from national surveys and/or qualitative information from partner organizations currently engaged in promoting food fortification programs in the eligible countries. It was assumed that WRA represent on average approximately 25% of a country’s population, as of 2019 [17].

*Step 4: Estimating the number of anemic WRA reached by fortified wheat flour/rice.* Country-specific WRA anemia prevalence estimates were applied to the number of WRA previously estimated (*see Step 3*) to quantify the number of anemic WRA reached through fortified grains [2].

*Step 5: Calculating the number of cases of anemia among WRA that could be averted as a result of large-scale fortification.* Documented impact of large-scale food fortification has shown increased serum ferritin in several populations and demonstrated a positive impact on functional outcomes, including a 34% reduction in anemia (relative risk (RR): 0.66; 95% confidence interval (CI): 0.59, 0.74) [7]. This effect size was applied to the number of anemic WRA reached through grain fortification to compute the cases of anemia among WRA that could be averted following large-scale fortification in the selected countries. 

### 2.2. Estimation Method for Number of Folic Acid-Preventable NTDs Prevented and Child Deaths Averted in Eligible Countries:

Steps 1 and 2 mentioned in Section 2.1 above were also used to determine the proportion of wheat flour/rice that is industrially milled but not currently fortified. The prevalence of folic acid-preventable NTDs in countries with immediate large-scale fortification potential was then estimated. 

*Step 3: Estimating annual number of live births.* The annual number of live births per country was obtained from the United Nations database of births for the latest year of data available at the time (2018) [18]. 

*Step 4: Estimating annual number of live births affected by NTDs.* Country-specific modeled estimates of the live birth prevalence of NTDs were obtained from the study published by Blencowe et al. (2018) [3]. These estimates are modeled pre-fortification prevalence estimates according to best available data. The country-specific annual number of NTDs was then estimated as the product of 2018 estimated live births taken from the United Nations database of births, and the prevalence of NTDs was taken from Blencowe et al. (2018) estimates. Using Blencowe et al. (2018) estimates, we also estimated the country-specific number of elective terminations and stillbirths with NTDs [3]. To estimate under-five child deaths and survivors to age five, the data from Blencowe et al. (2018) were again used [3]. 

*Step 5: Estimating post-fortification prevalence of NTDs.* The post-fortification birth prevalence of NTDs was empirically set at a rate of six per 10,000 births (including live and stillbirths), according to a study by Crider and colleagues (2014) and other published reports from countries with successful folic acid fortification programs that reported a similar NTD prevalence in their post-fortification NTD surveillance studies [19,20,21,22,23]. 

*Step 6: Calculating the annual number of folic acid-preventable NTDs averted per country.* On the basis of the estimated coverage of industrially milled rice/wheat flour with the potential to be fortified, the annual number of folic acid-preventable NTD cases averted per country was calculated as the difference in the number of cases according to a country’s pre-fortification prevalence number of cases expected post-fortification (i.e., six per 10,000 births). For example, if a country had a NTD prevalence of 25 per 10,000 births (pre-fortification), we first calculated the number of cases that would occur at this pre-fortification prevalence level, and then subtracted the number of cases that are expected at a post-fortification prevalence of six per 10,000 births.

### 2.3. Estimation Method for Number of DALYs Averted and the Economic Value of Health Benefits of Fortification at Scale

*Step 1: Estimating annual number of DALYs averted due to anemia in WRA.* The number of years living with disability (YLD) averted, which refers to the total morbidity averted due to anemia, was estimated. This was done by taking the product of the number of cases of anemia averted, the corresponding disability weight of anemia, and the duration of the effect of anemia prevention from consuming adequately fortified staple foods (assumed to be one year only). This calculation used previously derived results for burden of anemia (morbidity) at the country level for each mild, moderate, and severe category of anemia and corresponding disability weights for the matching severity category [24,25,26]. 

*Step 2: Estimating annual number of DALYs averted due to folic acid-preventable NTDs averted.* The years of life lost (YLL), which refers to the total number of years of expected life lost due to premature mortality from folic acid-preventable NTDs, and the number of the number of years living with disability (YLD) averted for children surviving past age five were estimated. The estimation of YLDs averted by averting folic acid-preventable NTDs was estimated as the product of the number of cases of averted NTDs, NTD-specific disability weights, and the expected remaining lifespan of affected children in each country as the duration of the effect of fortification on averting these cases. 

The estimation of YLLs averted due to NTDs averted was estimated as the product of the number of cases of folic acid-preventable NTD-related child deaths averted and the expected lifespan of the child in each country. The total number of DALYs averted is the sum of the YLD and YLL averted in each country.

*Step 3: Estimating the economic value of health benefits due to fortification at scale.* In order to estimate the approximate economic value of the burden of morbidity and mortality averted due to fortification, we undertook a monetized DALY approach using the value of a statistical life [27,28]. The estimation of economic value of morbidity and mortality averted due to fortification was estimated as the product of the number of DALYs averted and a country-specific value of a statistical life [29]. The resulting figure is an estimate of the potential economic value of health benefits generated by fortification at scale in terms of United States dollars (USD) at the country and aggregate levels. Since averting folic acid-preventable NTDs leads to survival and health benefits that extend far into the future of a child’s life, it is important to discount the monetary values of benefits in order to translate them into present value. Discounting of future health and economic impacts is conducted to reflect the opportunity costs of investing in now in nutrition interventions to yield benefits into the future. For the primary analysis in this paper, a commonly used 3% default discount rate on the number of DALYs averted was used, and alternative 0% and 5% discount rates were calculated for the sensitivity analysis [28]. The approximate benefit–cost ratio of global fortification at the aggregate level was estimated using a global unit cost [30].

## 3. Results

**Figure 1:**
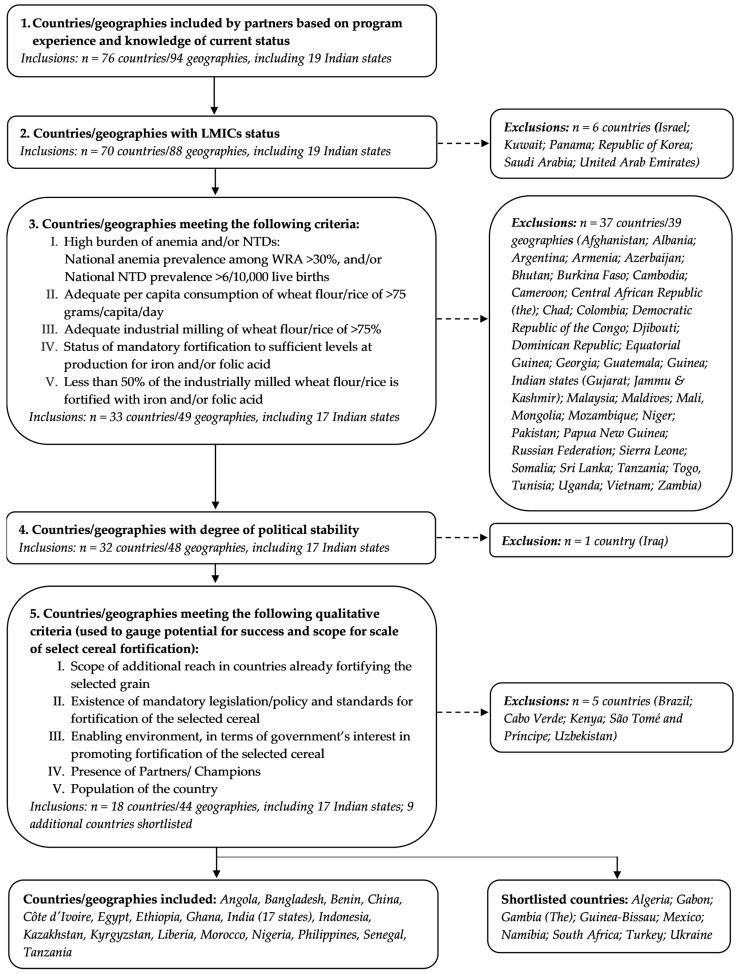
Flow diagram shows selection process to identify eligible countries with high impact potential for large-scale food fortification of wheat flour or rice.

The flow diagram shown in Figure 1 summarizes the process via which 18 LMICs with highest and most immediate potential for large-scale fortification of wheat flour and/or rice were identified. Starting with 76 countries recommended by the collaborating partners, six countries were excluded due to their high-income status, 37 were excluded when the quantitative criteria were applied, one country was excluded due to a perceived degree of political instability, and five were further excluded when the qualitative criteria were applied. This resulted in 18 priority countries of interest. Table 1 shows the 18 countries thus identified, namely, Angola, Bangladesh, Benin, China, Côte d’Ivoire, Egypt, Ethiopia, Ghana, India (17 states), Indonesia, Kazakhstan, Kyrgyzstan, Liberia, Morocco, Nigeria, Philippines, Senegal, and Tanzania. An additional nine countries were also shortlisted but were not further assessed for impact due to their small population size (<3 million people), additional contextual knowledge pertaining to political barriers, or uncertainty about existing fortification efforts. Table 1 summarizes the prevalence of anemia among WRA, NTD prevalence, per capita consumption of selected grains, and percentage availability of industrially milled cereal in each eligible country. Results for 17 Indian states are presented separately (Table A1), in view of their large population size, state regulations independent of a national regulation, and extent of social protection programs. A more detailed, qualitative analysis of country-specific reasons for selecting these 18 countries is presented in Table 2.

For wheat flour fortification, 12 LMICs were identified with an immediate potential for large-scale fortification with iron and/or folic acid. Of these countries, three have voluntary fortification (China, Ethiopia, and India), three do not have a mandate to fortify wheat flour with folic acid and/or iron (Angola, Bangladesh, and Egypt), three have legislation to mandate fortification of wheat flour with iron and/or folic acid but fortify less than 50% of industrially milled wheat flour (Kazakhstan, Kyrgyzstan, and Tajikistan), and three have a legislation to mandate but do not use iron and/or folic acid as a fortificant, do not use iron and/or folic acid in the globally recommended amounts, or do not use iron and/or folic acid in the globally recommended fortificant forms (Indonesia, Morocco, and Philippines) (Table 3). For rice fortification, nine LMICs were identified that have a high and immediate potential for fortification with iron and/or folic acid. Three of the nine LMICs identified also have a wheat flour fortification program. Of the nine countries, seven do not have a mandate to fortify rice with iron and/or folic acid (Benin, China, Côte d’Ivoire, Ghana, Liberia, Nigeria, and Senegal) and two voluntarily fortify rice (Bangladesh and India) (Table 4). 

In these 12 countries identified for wheat flour fortification, a total of 133.1 million metric tons of wheat flour is available for fortification, of which 7.1 million metric tons is fortified, and an additional 125.9 million metric tons of wheat flour can be fortified (Table 5). In the nine countries identified for rice fortification, a total of 141.3 million metric tons of rice was available for fortification, of which about 0.015 million metric tons was being fortified, and an additional 141.2 million metric tons of rice can be fortified (Table 5).

### 3.1. Number of Cases of Anemia Prevented among WRA

By fortifying industrially milled wheat flour and/or rice with iron in the 18 countries identified in the analysis, 72.1 million new cases of anemia among WRA could be averted annually (Table 6 and Table A1). That equates to an 11.8% reduction in the number of cases of anemia globally, and a 34.2% reduction of the burden of anemia in the 18 countries analyzed. 

### 3.2. Number of Cases of NTD Prevented and Under-Five Deaths Averted

By fortifying the industrially milled wheat flour and/or rice with folic acid in countries of interest, 51,636 new cases of folic acid-preventable NTDs could be averted (Table 6 and Table A1). That equates to a 38% reduction in the countries of interest. Additionally, 46,378 under-five child deaths associated with NTDs could be averted annually by fortification at scale. 

### 3.3. Value of Morbidity and Mortality Averted

The health and human capital impacts on the countries of interest are presented in Table 6. As a result of the estimated health impact of fortification at scale, there is the potential to avert 2.16 million YLD due to anemia prevented, 14,750 YLD due to folic acid-preventable NTDs averted, and 3.22 million YLL due to these averted cases. In aggregate, fortification has the potential to avert 5.4 million DALYs annually and generate an estimated economic value of 31.8 billion USD over the lifespan of women and children beneficiaries (Table 6, Table A2 and Table A3). A disproportionate amount of the total discounted economic value benefits China (63%), due to its high value of a statistical life and large population, and 64% of the aggregate economic value is associated with the prevention of anemia in women (at a 3% discount rate). Using an approximate global fortification unit cost of 0.37 USD per person reached, this translates into a mean benefit–cost ratio of 34:1 USD (range = 4–115:1 USD, median 20:1 USD) (Table A4 and Table A5) [32]. Even when doubling or tripling the unit cost per person reached in a sensitivity analysis (given variation in unit costs across countries and type of staple food), the mean benefit–cost ratio remains positive and high. 

## 4. Discussion

This analysis, conducted by a consortium of international experts and organizations, used the best available country-level data on the burden of anemia and NTDs, current fortification status, existing industrial milling opportunity, and amount of daily per capita wheat flour and/or rice available for human consumption to provide a comprehensive assessment of fortification potential in each country.

Eighteen LMICs were identified with the greatest potential for preventing anemia, folic acid-preventable NTDs, and associated under-five child deaths by implementing large-scale, mandatory fortification of wheat flour and/or rice with iron and folic acid. If implemented effectively and at scale across all 18 countries selected, 72.1 million cases of anemia among WRA, 51,636 cases of folic acid-preventable NTDs, and 46,378 child deaths among those born with NTDs could be averted. While the total numbers of potential cases of anemia and NTDs averted are highest in large countries such as China, there is a much higher potential for cases averted per capita in countries with a higher prevalence of anemia (i.e., Nigeria, Benin, Côte d’Ivoire, and India) or folic acid-preventable NTDs (i.e., Angola, India, Benin, and Nigeria). This magnitude and type of prevention has lifelong health and economic implications and would make a significant contribution to reaching the health and equity 2030 SDGs. This study estimated that fortification at scale has an economic value of 31.8 billion USD per year and yields a positive benefit–cost ratio (with expected variation between countries) that is consistent with previous cost–benefit analyses in the literature [33,34]. This analysis did not estimate the benefit of preventing folate deficiency anemia; therefore, the health and economic benefits could be considered conservative. Detailed, country-level health and economic evaluation is recommended.

Large-scale food fortification conveys equitable benefits across populations that consume fortifiable food grains. Fortification is proven to be one of the most successful and cost-effective public health programs that countries can invest in, using minimal resources and funds [35]. Furthermore, the benefits of fortification compound annually as anemia, NTDs, and associated morbidity and mortality are averted each year. Once industrial mills begin fortification as part of standard operating procedures, the same technology can be used to include other vitamins and minerals (e.g., zinc, vitamin B_12_, and other B vitamins) and address multiple nutritional deficiencies without substantial additional expenses. 

This review purposely chose food staples such as wheat flour and rice that are regularly and widely consumed by a large portion of population groups in respective countries to allow prioritization as a function of maximum reach and impact potential. It further sought to ensure that all segments of the population will benefit from fortified foods due to the low cost required to add micronutrients. This is particularly the case for urban slum areas and through social safety nets where centrally processed fortified foods reach the poorest and most vulnerable.

Barriers to the implementation of successful, large-scale mandatory food fortification programs have been documented and can be multi-faceted, including political instability, lack of political support leading to under-prioritization of fortification by the government, absence of incentives for food industry, food industry’s lack of capacity and resources, and ineffective and weak regulation and enforcement [36]. Efforts required to overcome important hurdles and achieve long-term success include more effective communication around the cost savings (human and financial) of fortification at the country level and shifts in political commitment for initiating and sustaining a program. Furthermore, it is recommended that stakeholders assess barriers by conducting a thorough landscape assessment of enabling and disabling factors including the presence (or absence) of champions in-country who could drive a fortification agenda and promote sustained government commitment [37]. The proposed countries in our review were given careful consideration to these factors to ensure a high potential for success.

Although the 18 countries identified in this study are those that were found to have the most immediate impact on prevention of anemia among WRA and NTDs, this does not mean that the excluded countries, including those that are high-income and those that have a degree of political instability, would not also benefit from strong and sustained technical support. Socio-economic and health disparities within high-income countries should not be discounted. Although the authors chose a country’s income status as a criterion, another approach could have been to stay agnostic to income status while highlighting the preventable health disparities within and between countries, particularly in light of the number of high-income countries that fail to mandate fortification. Additionally, the 76 countries with which the analysis started were based on collaborating partners’ subjective, expert recommendations gleaned from years of sustained efforts with government counterparts and industry in the countries analyzed. In the future, a comprehensive global analysis would allow for a more detailed comparison between this paper’s included and excluded countries. Our prevention estimates for anemia and folic acid-preventable NTDs may be conservative as they are aggregated across multiple settings and derived from modeled extrapolations. Therefore, actual impact could be higher than estimated. While estimating impact of food fortification on anemia, benefits reaching non-WRA were not calculated. Similarly, prevention of folic acid-preventable NTDs among early spontaneous abortions could not be estimated.

Despite its limitations, this analysis has several strengths. It is timely, relevant, and actionable. The analysis used best available data and prevalence estimates for modeling health impact as a result of scaling up wheat flour and rice fortification programs. The analysis was compiled by leaders in the fields of nutrition, food fortification, and epidemiology of NTDs who collaborated to present this comprehensive review of fortification in select countries. Factors included in this prioritization exercise are established, industry-relevant criteria for fortification at scale.

## 5. Conclusions

Effective fortification programs are needed in countries that demonstrate high rates of anemia among WRA due to iron deficiency and high occurrence of NTDs due to maternal folate deficiency before and during early pregnancy. 

Where there is a demonstrated need and appropriate food vehicle(s) for effective delivery of key micronutrients, much progress has been made globally to ensure national mandatory fortification programs are put in place. However, there are still many countries that could benefit from the health and economic gains of fortification that are yet to implement them effectively and at scale [36]. The LMICs identified in our analysis have strong, already-established industrial milling infrastructures and high coverage and consumption of the respective grain, coupled with some of the world’s heaviest nutritional burdens. Each of the countries of interest analyzed in the study is poised and ready to initiate and adopt large-scale grain fortification programs. This presents a unique opportunity to achieve marked reductions in the prevalence of anemia among WRA and folic acid-preventable NTDs, which are major contributors to individual growth and development, the burden of disease, healthcare costs, and barriers to economic productivity. This opportunity warrants an urgent call to action for stronger political support among government leaders, key stakeholders in each of these countries, and global and regional technical support bodies that more can and should be done now to save lives, improve quality of life, and increase economic productivity at a national and global scale through the adoption and maintenance of strong and sustained fortification programs. Strong, sustained fortification programs may give countries an opportunity to achieve at least nine of the 17 SDGs, namely, (1) no poverty, (2) zero hunger, (3) good health and well-being, (4) quality education, (5) gender equality, (8) decent work and economic growth, (10) reduced inequalities, (11) sustainable cities and communities, and (17) partnership for the goals.

## Figures and Tables

**Table 1:** Countries identified for their potential to maximize reduction in anemia and neural tube defects (largely comprising folic acid-preventable spina bifida, anencephaly, and encephalocele) through large-scale fortification of wheat flour/rice with iron and/or folic acid.

No.	Country	Anemia among WRA and/or NTD Prevalence		Wheat Flour		Rice	
				Per Capita Consumption	% Availability of Industrially Milled Grains	Per Capita Consumption	% Availability of Industrially Milled Grains
		>30% Anemia Prevalence among Nonpregnant WRA	>6 NTDs /10,000 Live Births (LBs)	≥75 g/c/day	≥75% (Considering Imports to Be Industrially Milled)	≥75 g/c/day	≥75% (Considering Imports to Be Industrially Milled)
		**Source:** Most recent Demographic Health Surveys/National Micronutrient Surveys/WHO Global Observatory 2016	**Source:** Blencowe 2018 estimates (personal communication)	**Source:** Global Fortification Data Exchange (GFDx)	**Source:** FFI Country Profile January 2020	**Source:** Global Fortification Data Exchange (GFDx)	**Source:** FFI Country Profile January 2020
1	Angola	47.7%	15.3	112	100%		
2	Bangladesh	26%	32.0	48	73% **	470	60% **
3	Benin	57.7%	15.3			146	83%
4	China	26.4%	19.4	174	89%	214	90%
5	Côte d’Ivoire	53.7%	9.9			174	30% **
6	Egypt	25.2%	17.5	402	100%		
7	Ethiopia	23.6%	15.3	86	55% **		
8	Ghana	42.4%	15.3			88	53% **
9	India (17 states) *	53.1%	30.0	140	*	184	*
10	Indonesia	26%	13.1	70 **	100%		
11	Kazakhstan	30.7%	9.9	253	100%		
12	Kyrgyzstan	35.2%	9.9	354	85%		
13	Liberia	44.5%	15.3			260	49% **
14	Morocco	36.9%	17.5	492	100%		
15	Nigeria	57.8%	15.3			77	47% **
16	Philippines	11.8%	13.1	63 **	100%		
17	Senegal	54.1%	9.9			198	68% **
18	Tajikistan	41.1%	9.9	358	60% **		

* India was included in consideration of the impact of reaching a large number of people in the eligible 17 Indian states with fortified wheat flour/rice under the country’s largest social protection program, the Public Distribution System, which reaches over 60% of the population. ** Country-specific reasons for selecting countries that did not meet the defined criteria are mentioned in Table 2. FFI: Food Fortification Initiative; GFDx: Global Fortification Data Exchange; LB: live births; NTDs: neural tube defects; No.: number; WHO: World Health Organization; WRA: women of reproductive age. Cells in gray color shade indicate that these countries were not selected for fortification of given food vehicle.

**Table 2:** Country-specific reasons for selecting 18 low- and middle-income countries with high potential for large-scale fortification of wheat flour/rice.

**A. Strong political will and/or reach opportunity**	
Bangladesh and India	The governments of Bangladesh and India have demonstrated strong political will for fortification through the adoption of voluntary fortification of wheat flour and rice, and the countries are committed to the integration of these fortified vehicles into national social safety net programs. This presents an immediate opportunity to reach the most vulnerable populations at scale. India has the largest number of pregnancies in the world affected by NTDs and presents an opportunity to reach 400 million people with fortified wheat flour/rice.
China	330,000 pregnancies are affected by NTDs each year; this is 13% of the global total and second only to India in the number of pregnancies affected [3]. With such a significant burden, there is high potential in China to prevent NTDs through folic acid fortification.
Côte d’Ivoire	The government is leading the way in the West Africa region by considering the introduction and scale-up of rice fortification, including fortification of imported rice that constitutes ~30% of total rice available for consumption [16].
Egypt	Egypt is yet another case exhibiting strong political support and potential for expansive reach. The government is currently working to restart the country’s social safety net wheat flour fortification program with interest in expanding it into the open market. Fortified wheat flour provided through the social safety net program alone would reach over 73 million vulnerable people. If fortified wheat flour was provided through both the social safety net program and the open market, 90% of the population would be reached (Food Fortification Initiative, personal communication).
Ethiopia	Although only 55% of Ethiopia’s wheat flour is milled industrially and the coverage of fortifiable wheat flour in Ethiopia is only 28% [31], this country was included due to the relatively large population of Ethiopia (104.9 million) and high rate of NTDs (130 per 10,000 births) [32]. Coverage of only 28% in this country means that almost 30 million people can be reached with fortified foods. Additionally, the government has adopted voluntary fortification of wheat flour, exhibiting strong political will to address micronutrient deficiencies through fortification. Lastly, there are multiple champions to support fortification of wheat flour with folic acid in the country.
**B. Opportunity for fortifying imports**	
Angola Bangladesh Benin Côte d’Ivoire Ghana Indonesia Liberia Philippines Senegal	Imported wheat flour and/or rice remain a dominant staple, presenting an opportunity for feasible and effective grain fortification in half of the eligible countries (9 out of 18). By mandating fortification of imported wheat flour/rice, these countries could reap large public health benefits while facilitating an enabling environment for other countries in the region seeking similar mandates. A considerable proportion of wheat flour that is available for consumption is imported in Angola (>90%), Philippines, (>90%), Indonesia (>90%), and Bangladesh (>70%). The same holds true for rice in Benin (>90%), Senegal (>70%), Côte d’Ivoire (>50%), Ghana (>50%), and Liberia (>50%).
Nigeria	Nigeria consumes more rice than any other country in Africa and, although only ~37% of the rice available for consumption is imported [16], the large population (190.8 million), high burden of anemia among women of reproductive age (47%), and high prevalence of neural tube defects (15/10,000 live births) make it a tremendous opportunity for rice fortification.
**C. Support required due to technical limitations and constrained resources (market, economic, and behavior)**	
KazakhstanKyrgyzstanMoroccoPhilippinesTajikistan	Kazakhstan, Kyrgyzstan, Morocco, Philippines, and Tajikistan have all expressed strong political will by legislating fortification of wheat flour. While Tajikistan is the newest country to legislate wheat flour fortification in 2018 and would benefit from support, suboptimal implementation of the legislation in Kazakhstan, Kyrgyzstan, Morocco, and the Philippines is hindering the achievement of optimal health impact in these countries. Support for the industry to adequately fortify wheat flour and for the government to enforce monitoring and compliance to the national fortification standards in these countries will go a long way in ensuring a sustainable wheat flour fortification intervention. In the Philippines, simply adding folic acid to the already-existing wheat flour/rice fortification standards and enforcing its implementation will help the country address the high burden of NTDs.

NTDs: neural tube defects.

**Table 3:** Eligible countries that could have a high potential impact through wheat flour fortification: information pertaining to legislative status and inclusion of iron and folic acid in fortification standards.

No.	Eligible Country	Wheat Flour Fortification					
		Legislation	Iron			Folic Acid (ppm)	
			Level (ppm)	Compound Indicated in Standard	Level (ppm) as Per Global Recommendation for Country’s Cereal Grain Consumption	Level (ppm)	Level (ppm) as Per Global Recommendation for Country’s Cereal Grain Consumption
1	Angola *	Unknown	No fortification standard		40 as NaFeEDTA *** 60 as FF/FS ***	No fortification standard	2.6
2	Bangladesh **	Unknown	55	Not specified	40 as NaFeEDTA *** 60 as FF/FS ***	2	5
3	China *	Voluntary 2012	20	No information	20 as NaFeEDTA *** 30 as FF/FS *** 60 as EI ***	2	1.3
4	Egypt *	Unknown	No fortification standard		15 as NaFeEDTA *** 20 as FF/FS *** 40 as EI ***	No fortification standard	1
5	Ethiopia **	Voluntary 2017	30 40	NaFeEDTA *** FF ***	40 as NaFeEDTA *** 60 as FF/FS ***	2	2.6
6	India (6 states) **^#^	Voluntary 2018	14–21.25 28–42.5	NaFeEDTA *** FS/FF/EI/FC/FP/FB/FL ***	20 as NaFeEDTA ***	0.075-0.125	1.3
7	Indonesia **	Mandatory 2001	50	NaFeEDTA/FF/FS ***	40 as NaFeEDTA *** 60 as FF/FS ***	2	5
8	Kazakhstan *	Mandatory 2005	55	-	20 as NaFeEDTA *** 30 as FF/FS *** 60 as EI ***	1.4	1.3
9	Kyrgyzstan *	Mandatory 2009	No fortification standard		15 as NaFeEDTA *** 20 as FF/FS *** 40 as EI ***	No fortification standard	1
10	Morocco *	Mandatory 2005	45	No information	15 as NaFeEDTA *** 20 as FF/FS *** 40 as EI ***	1.53	1
11	Philippines *	Mandatory 2000	87.5	No information	40 as NaFeEDTA *** 60 as FF/FS ***	0	5
12	Tajikistan *	Mandatory 2019	No fortification standard		15 as NaFeEDTA *** 20 as FF/FS *** 40 as EI ***	No fortification standard	1

Source of Information: * Global Fortification Data Exchange, https://fortificationdata.org/, accessed 16 April 2020. ** Country fortification standards document. *** NaFeEDTA: sodium iron ethylenediaminetetraacetate; FF: ferrous fumarate; FS: ferrous sulfate; EI: electrolytic iron; FC: ferrous citrate; FL: ferrous lactate; FB: ferrous bisglycinate; ppm: parts per million. ^#^ Six selected states in India include Haryana, Himachal Pradesh, Madhya Pradesh, Punjab, Rajasthan, and Uttar Pradesh.

**Table 4:** Eligible countries that could have a high potential impact through rice fortification: information pertaining to legislative status and inclusion of iron and folic acid in fortification standards.

No.	Eligible Country	Rice Fortification					
		Legislation	Iron			Folic Acid (ppm)	
			Level (ppm)	Compound Indicated in Standard	Level (ppm) as Per Global Recommendation for Country’s Cereal Grain Consumption	Level (ppm)	Level (ppm) as Per Global Recommendation for Country’s Cereal Grain Consumption
1	Bangladesh **	Voluntary 2015	60	Micronized ferric pyrophosphate	70 as micronized ferric pyrophosphate	1.7	1
2	Benin *	None	No fortification standard		120 as micronized ferric pyrophosphate	No fortification standard	2.6
3	China *	None	No fortification standard		70 as micronized ferric pyrophosphate	No fortification standard	1.3
4	Côte d’Ivoire	None	No fortification standard		70 as micronized ferric pyrophosphate	No fortification standard	1.3
5	Ghana *	None	No fortification standard		120 as micronized ferric pyrophosphate	No fortification standard	2.6
6	India (11 states) ^#^	Voluntary 2018	28–42.5 14–21.25	Ferric pyrophosphateNaFeEDTA	70 as micronized ferric pyrophosphate	0.075–0.125	1.3
7	Liberia *	None	No fortification standard		70 as micronized ferric pyrophosphate	No fortification standard	1.3
8	Nigeria *	None	No fortification standard		120 as micronized ferric pyrophosphate	No fortification standard	2.6
9	Senegal **	None	No fortification standard		70 as micronized ferric pyrophosphate	No fortification standard	1.3

Source of information: * Global Fortification Data Exchange, https://fortificationdata.org/, accessed 16 April 2020. ** Country fortification standards document. NaFeEDTA: ferric sodium ethylenediaminetetraacetate; ppm: parts per million. ^#^ 11 selected states in India include Andhra Pradesh, Assam, Bihar, Chhattisgarh, Jharkhand, Karnataka, Kerala, Maharashtra, Orissa, Tamil Nadu, and West Bengal.

**Table 5:** Proportion and amount of industrially milled wheat flour and rice available for fortification in each eligible country.

No.	Eligible Country	Wheat Flour						Rice					
		Proportion of Industrially Milled Wheat Flour Available Post Harvest/Processing Available for consumption (%)—To Achieve Scale	Total Industrially Milled Wheat Flour Available Post Harvest/Processing Available for Consumption (MT)	Proportion of Industrially Milled Wheat Flour Available That Is Already Fortified (%)	Total Industrially Milled Wheat Flour Available That Is Already Fortified (MT)	Proportion of Industrially Milled Wheat Flour Available with Potential to be Fortified (%)	Total Industrially Milled Wheat Flour Available with Potential to be Fortified (MT)	Proportion of Industrially Milled Rice Available Post Harvest/Processing Available for Consumption (%)—To Achieve Scale	Total Industrially Milled Rice Available Post Harvest/Processing Available for Consumption (MT)	Proportion of Industrially Milled rice Available That Is Already Fortified (%)	Total Industrially Milled Rice Available That Is Already Fortified (MT)	Proportion of Industrially Milled Rice Available with Potential to be Fortified (%)	Total Industrially Milled Rice Available with Potential to be Fortified (MT)
1	Angola	70%	368,972	0%	-	70%	368,972						
2	Bangladesh	55%	2,559,890	0.5%	25,599	54.5%	2,534,291	*	750,000	2%	15,000	*	735,000
3	Benin							97%	1,374,002	0%	-	97%	1,374,002
4	China	63%	80,697,739	1%	806,977	62%	79,890,761	90%	113,060,636	0%	-	90%	113,060,636
5	Côte d’Ivoire							40%	779,045	0%	-	40%	779,045
6	Egypt	70%	13,782,824	0%	-	70%	13,782,824						
7	Ethiopia	48%	2,654,994	0%	-	48%	2,654,994						
8	Ghana							65%	644,328	0%	-	65%	644,328
9	India (17 states)	*	12,853,149	0%	-	*	12,853,149	*	20,330,033	0%	-	*	20,330,033
10	Indonesia	70%	4,813,060	61%	4,187,362	9%	625,698						
11	Kazakhstan	70%	4,943,239	29%	2,026,728	41%	2,916,511						
12	Kyrgyzstan	63%	838,878	4%	58,721	59%	780,157						
13	Liberia							62%	264,208	0%	-	62%	264,208
14	Morocco	70%	6,656,713	0%	-	70%	6,656,713						
15	Nigeria							54%	3,076,626	0%	-	54%	3,076,626
16	Philippines	70%	1,857,509	0%	-	70%	1,857,509						
17	Senegal							80%	1,035,376	0%	-	80%	1,035,376
18	Tajikistan	56%	1,023,695	0%	-	56%	1,023,695						
Total			133,050,662		7,105,387		125,945,275		141,314,254		15,000		141,299,254

MT: metric tons; No.: number. * In both India and Bangladesh, the amount of industrially milled staple food required to reach registered beneficiaries under social safety net programs was estimated instead of the overall proportion of industrially milled staple food available for consumption through open market channels. Cells in gray color shade indicate that these countries were not selected for fortification of given food vehicle.

**Table 6:** Estimated annual health and economic benefits of fortification at scale in countries of interest.

Country	Number of People Reached (Millions)	Cases of Anemia in WRA Averted	Cases of NTDs Averted	Child Deaths Averted	DALYs Averted	Economic Value of DALYs Averted (in Millions of USD)
Angola	9.0	365,698	816	769	57,522	383
Bangladesh	144.6	3,194,605	4154	3857	369,392	594
Benin	10.3	503,202	375	354	38,998	64
China	1284.8	28,831,810	14,037	11,681	1,701,396	20,298
Côte d’Ivoire	12.3	559,521	142	134	26,294	93
Egypt	88.6	1,896,955	2077	1783	161,352	699
Ethiopia	84.5	1,695,531	1397	1315	140,088	141
Ghana	20.0	722,468	531	499	54,008	147
India (17 states)	553.1	24,950,107	22,006	20,410	2,198,103	5532
Indonesia	24.5	540,840	312	274	35,071	196
Kazakhstan	16.5	429,785	62	53	16,768	370
Kyrgyzstan	5.7	169,646	36	29	7666	17
Liberia	2.8	105,235	92	87	8555	6
Morocco	32.4	1,016,226	547	454	66,193	310
Nigeria	109.4	5,374,530	3731	3519	362,564	2364
Philippines	80.7	809,657	1089	954	102,517	537
Senegal	14.2	653,907	171	161	32,183	64
Tajikistan	7.8	273,501	61	47	11,371	29
Total	2.5 B	72.1 M	51,636	46,378	5.4 M	31.84 B

DALYs: disability adjusted life years; NTDs: neural tube defects; USD: United States dollar; WRA: women of reproductive age; B: billion; M: million.

## Data Availability

Multiple public and third party datasets were used. Publicly available datasets analyzed in this study can be found here: (a) World Health Organization. *The Global Prevalence of Anaemia in 2011*; World Health Organization: Geneva, Switzerland, 2015. World Health Organization. *The Global Prevalence of Anaemia in 2011*; World Health Organization: Geneva, Switzerland, 2015. Available online: https://www.who.int/nutrition/publications/micronutrients/global_prevalence_anaemia_2011/en/. (b) Global Fortification Data Exchange (GFDx). Available online: https://fortificationdata.org. (c) UNICEF. State of the World’s Children 2019. Available online: https://www.unicef.org/media/63016/file/SOWC-2019.pdf. (d) The Food Fortification Initiative. Country Profiles. Available online: https://www.ffinetwork.org/country-profiles. (e) Global Burden of Disease Collaborative Network. *Global Burden of Disease Study 2017 (GBD 2017) Disability Weights*; Institute for Health Metrics and Evaluation (IHME): Seattle, WA, USA, 2018. Available online: http://ghdx.healthdata.org/record/ihme-data/gbd-2017-disability-weights. (f) World Health Organization. Global Health Observatory Data Repository; Prevalence of Anaemia in Women of Reproductive Age; WHO: Geneva, Switzerland, 2017. Available online: https://apps.who.int/gho/data/view.main.ANAEMIAWOMENPREVANEMIAREG?lang=en Viscusi, W.K.; Masterman, C.J. Income Elasticities and Global Values of a Statistical Life. *J. Benef. Cost Anal.* 2017. Available online: https://static1.squarespace.com/static/5be33b0efcf7fdd77c7823be/t/5be9f13021c67c13124b3a77/1542058289675/361_Income_Elasticity_of_Global_Values_of_a_Statistical_Life.pdf. (g) ICF, 2012. The DHS Program STATcompiler. Funded by USAID. Available online: http://www.statcompiler.com. Restrictions apply to the availability of these data. Data was obtained from Blencowe et al., (2018) [3] and are available from the authors with the permission of Dr. Hannah Blencowe.

## Appendix A

**Table A1:** Estimated annual health impact and economic value of fortification at scale: 17 Indian states.

Indian State	People Reached (Millions)	Cases of Anemia in WRA Averted	Cases of NTDs Averted	Child Deaths Averted	DALYs Averted	Economic Value of DALYs Averted (in USD $ m)
Andhra Pradesh	25.1	1,282,143	943	874	100,154	260
Assam	16.4	639,396	816	757	72,946	172
Bihar	56.2	2,882,247	2364	2193	240,692	612
Chhattisgarh	13.5	539,333	800	742	68,509	157
Haryana	13.2	702,042	617	572	61,084	153
Himachal Pradesh	3.5	158,175	117	108	12,472	33
Jharkhand	17.6	975,552	806	748	81,499	206
Karnataka	31.4	1,195,893	1266	1174	120,029	293
Kerala	16.8	490,413	580	538	54,086	131
Madhya Pradesh	38.8	1,732,766	1691	1569	163,140	403
Maharashtra	57.4	2,341,889	2084	1933	208,893	527
Orissa	21.6	934,548	798	740	80,870	205
Punjab	14.1	640,540	481	446	51,056	133
Rajasthan	36.9	1,466,494	1946	1805	171,827	401
Tamil Nadu	36.4	1,701,949	1093	1013	123,627	331
Uttar Pradesh	107.8	4,800,876	4085	3788	413,783	1049
West Bengal	46.4	2,465,850	1519	1409	173,436	466
**TOTAL (17 states)**	**553.1**	**24,950,107**	**22,006**	**20,410**	**2,198,103**	**5532**

**Table A2:** Sensitivity analysis: monetized DALYs at 0%, 3%, and 5% discount rates at a national level.

Country/ Region	Monetized DALYs ($ Millions)		
	0% Discount Rate	3% Discount Rate	5% Discount Rate
Angola	676.75	383.2	303.4
Bangladesh	1042.42	593.7	486.3
Benin	91.26	63.5	56.1
China	29,863.50	20,298.0	18,150.7
Côte d’Ivoire	110.28	93.4	88.6
Egypt	1284.55	699.3	559.0
Ethiopia	214.26	140.7	121.9
Ghana	214.88	146.7	128.7
India (17 states)	8669.83	531.5	4756.8
Indonesia	286.79	195.6	173.6
Kazakhstan	430.41	370.1	356.0
Kyrgyzstan	20.77	17.4	16.6
Liberia	8.68	5.6	4.8
Morocco	448.41	309.9	278.3
Nigeria	3220.71	2363.9	2112.4
Philippines	864.40	536.7	457.1
Senegal	79.11	64.1	60.3
Tajikistan	34.57	28.6	27.2
**Total**	**47.6 B**	**31.8 B**	**28.1 B**

**Table A3:** Sensitivity analysis: monetized DALYs at 0%, 3%, and 5% discount rates: 17 Indian states

Indian State	Monetized DALYs ($ millions)		
	0% Discount Rate	3% Discount Rate	5% Discount Rate
Andhra Pradesh	394.76	260.3	227.2
Assam	288.1	171.7	142.9
Bihar	949.14	612	528.7
Chhattisgarh	270.73	156.7	128.6
Haryana	240.95	153	131.3
Himachal Pradesh	49.16	32.5	28.4
Jharkhand	321.41	206.4	178
Karnataka	473.71	293.2	248.6
Kerala	213.5	130.8	110.3
Madhya Pradesh	643.72	402.5	343
Maharashtra	823.89	526.6	453.2
Orissa	318.92	205.1	177
Punjab	201.24	132.7	115.7
Rajasthan	678.75	401.2	332.7
Tamil Nadu	486.96	331.1	292.6
Uttar Pradesh	1631.79	1049.20	905.4
West Bengal	683.09	466.5	413
**TOTAL**	**8.7 B**	**5.5 B**	**4.8 B**

**Table A4:** Sensitivity analysis: cost–benefit ratio at 0%, 3%, and 5% and 1× and 3× unit cost (0.37 USD) at a national level.

Country/Region	Benefit-Cost Ratio					
	0% Discount Rate		3% Discount Rate		5% Discount Rate	
	At 1× Unit Cost	At 3× Unit Cost	At 1× Unit Cost	At 3× Unit Cost	At 1× Unit Cost	At 3× Unit Cost
Angola	202.79	67.60	114.81	38.27	90.93	30.31
Bangladesh	19.49	6.50	11.10	3.70	9.09	3.03
Benin	24.04	8.01	16.74	5.58	14.78	4.93
China	62.82	20.94	42.70	14.23	38.18	12.73
Côte d’Ivoire	24.32	8.11	20.60	6.87	19.54	6.51
Egypt	39.20	13.07	21.34	7.11	17.06	5.69
Ethiopia	6.85	2.28	4.50	1.50	3.90	1.30
Ghana	28.97	9.66	19.78	6.59	17.35	5.78
India (17 states)	42.37	14.12	27.03	9.01	23.25	7.75
Indonesia	31.67	10.56	21.61	7.20	19.17	6.39
Kazakhstan	70.63	23.54	60.74	20.25	58.42	19.47
Kyrgyzstan	9.90	3.30	8.32	2.77	7.93	2.64
Liberia	8.43	2.81	5.46	1.82	4.68	1.56
Morocco	37.40	12.47	25.85	8.62	23.22	7.74
Nigeria	79.57	26.52	58.40	19.47	52.19	17.40
Philippines	28.94	9.65	17.97	5.99	15.30	5.10
Senegal	15.03	5.01	12.19	4.06	11.47	3.82
Tajikistan	11.93	3.98	9.89	3.30	9.39	3.13
**TOTAL**	**51.40**	**17.13**	**34.41**	**11.47**	**30.41**	**10.14**
**MEDIAN**	28.96	9.65	20.19	6.73	17.20	5.73
**RANGE**	6.9–202.8	2.3–67.6	4.5–114.8	1.5–38.3	3.9–90.9	1.3–30.3

**Table A5:** Sensitivity analysis: cost–benefit ratio at 0%, 3%, and 5% and 1× and 3× unit cost (0.37 USD): 17 Indian states.

Country/Region	Benefit-Cost Ratio					
	0% Discount Rate		3% Discount Rate		5% Discount Rate	
	At 1× Unit Cost	At 3× Unit Cost	At 1× Unit Cost	At 3× Unit Cost	At 1× Unit Cost	At 3× Unit Cost
Andhra Pradesh	42.44	14.15	27.99	9.33	24.42	8.14
Assam	47.62	15.87	28.37	9.46	23.62	7.87
Bihar	45.62	15.21	29.41	9.8	25.41	8.47
Chhattisgarh	54.2	18.07	31.37	10.46	25.74	8.58
Haryana	49.44	16.48	31.4	10.47	26.94	8.98
Himachal Pradesh	38.2	12.73	25.27	8.42	22.08	7.36
Jharkhand	49.35	16.45	31.69	10.56	27.33	9.11
Karnataka	40.77	13.59	25.23	8.41	21.39	7.13
Kerala	34.3	11.43	21.01	7	17.73	5.91
Madhya Pradesh	44.81	14.94	28.02	9.34	23.87	7.96
Maharashtra	38.79	12.93	24.8	8.27	21.34	7.11
Orissa	39.98	13.33	25.72	8.57	22.19	7.4
Punjab	38.61	12.87	25.45	8.48	22.2	7.4
Rajasthan	49.76	16.59	29.41	9.8	24.39	8.13
Tamil Nadu	36.15	12.05	24.58	8.19	21.73	7.24
Uttar Pradesh	40.92	13.64	26.31	8.77	22.7	7.57
West Bengal	39.77	13.26	27.16	9.05	24.05	8.02
**TOTAL**	**42.37**	**14.12**	**27.03**	**9.01**	**23.25**	**7.75**
**MEDIAN**	40.92	13.64	27.16	9.05	23.62	7.87
**RANGE**	34.3–54.2	11.4–18.1	21–31.7	7–10.6	17.7–27.3	5.9–9.1
